# Utilization of the Drug–Polymer Solid Dispersion Obtained by Ball Milling as a Taste Masking Method in the Development of Orodispersible Minitablets with Hydrocortisone in Pediatric Doses

**DOI:** 10.3390/pharmaceutics16081041

**Published:** 2024-08-04

**Authors:** Monika Trofimiuk, Katarzyna Olechno, Emil Trofimiuk, Anna Czajkowska-Kośnik, Patrycja Ciosek-Skibińska, Klaudia Głowacz, Joanna Lenik, Anna Basa, Halina Car, Katarzyna Winnicka

**Affiliations:** 1 Department of Clinical Pharmacy, Medical University of Bialystok, Mickiewicza 2a, 15-222 Bialystok, Poland; 2 Department of Pharmaceutical Technology, Medical University of Bialystok, Mickiewicza 2c, 15-222 Bialystok, Poland; anna.czajkowska-kosnik@umb.edu.pl (A.C.-K.); katarzyna.winnicka@umb.edu.pl (K.W.); 3 Department of Clinical Pharmacology, Medical University of Bialystok, Waszyngtona 15a, 15-274 Bialystok, Poland; emil.trofimiuk@umb.edu.pl (E.T.); halina.car@umb.edu.pl (H.C.); 4 Chair of Medical Biotechnology, Faculty of Chemistry, Warsaw University of Technology, Noakowskiego 3, 00-664 Warsaw, Poland; patrycja.ciosek@pw.edu.pl (P.C.-S.); klaudia.glowacz.dokt@pw.edu.pl (K.G.); 5 Department of Analytical Chemistry, Institute of Chemical Sciences, Faculty of Chemistry, Maria Curie Sklodowska University, Maria Curie-Sklodowska Square 3, 20-031 Lublin, Poland; joanna.lenik@mail.umcs.pl; 6 Faculty of Chemistry, University of Białystok, Ciołkowskiego 1K, 15-245 Białystok, Poland; abasa@uwb.edu.pl

**Keywords:** minitablets, hydrocortisone, hypromellose, solid dispersion, taste masking, ball milling

## Abstract

The objective of the conducted research was to design 2 mm orodispersible minitablets of pediatric doses of hydrocortisone (0.5 mg; 1.0 mg) with desirable pharmaceutical properties and eliminate the sensation of a bitter taste using preparation of solid dispersion by ball mill. Hydrocortisone was selected as the model substance, as it is widely utilized in the pediatric population. ODMTs were prepared by compression (preceded by granulation) in a traditional single-punch tablet machine and evaluated using pharmacopoeial tests, DSC, and FTIR analysis. The methods used to evaluate the effectiveness of the taste-masking effect included in vivo participation of healthy volunteers, in vitro drug dissolution and utilization of an analytical device—“electronic tongue”. The research employed a preclinical animal model to preliminary investigate the bioequivalence of the designed drug dosage form in comparison to reference products. The study confirmed the possibility of manufacturing good-quality hydrocortisone ODMTs with a taste-masking effect owing to the incorporation of a solid dispersion in the tablet mass.

## 1. Introduction

The acceptability of medicinal products is a pivotal issue to ensure adherence and therapeutic outcomes. Advanced pharmaceutical technology focuses on the evolution of patient-centric drug formulations in doses that are tailored to the needs of all age groups. The selection of the appropriate one is an essential and even mandatory prerequisite for providing effective pharmacotherapy since it is intended to facilitate the patient’s compliance, which is of particular importance with regard to children [1,2,3]. The significance of the acceptability of drug preparations has been emphasized by the International Council for Harmonisation of Technical Requirements for Pharmaceuticals for Human Use (ICH) in the guidelines concerning Good Clinical Practice (ICH-GCP) Q8 [4]. It should be emphasized that pharmacotherapy in pediatric patients is an issue that receives special attention, and the development of drug dosage forms designed for children is associated with many difficulties. The main problems arise from the heterogeneity of the pediatric population, as well as from the relatively small number of available drug dosage forms and appropriate dose selection. It is a widely known observation that children are not miniatures of adults, and they require a relevant dosing regimen rather than a proportional reduction of an adult’s dose. The dose adjustment is usually considerably more complex than simply reducing the one determined for adults. Therefore, light ought to be shed on the dose selection considering the age-specific alterations in pharmacokinetics and pharmacodynamics [5,6,7,8,9]. It is still prevalent that medicines intended for adult therapy, i.e., tablets and capsules, are used in pediatric patients’ treatment both at home and in pediatric care units. Adjusting the dose of traditional tablets is often performed by splitting or crushing, which poses the risk of inaccurate dosing, and thus inappropriate therapeutic levels, modifying the pharmacokinetics of the active substance and the occurrence of adverse effects [10,11,12,13].

A novel alternative to conventional tablets intended to be administered to patients of all ages are minitablets enabling individualized dosage adjustment (“by multiplication”) and ease of application. Minitablets represent a solid drug dosage form, which, owing to their size (1–3 mm), combine the advantages of the liquid formulations (flexibility of dosage, ease of swallowing) with the benefits of the solids (taste-masking, stability) [14,15,16,17,18]. Worth highlighting is the fact that minitablets have been indicated as a novel and needed pediatric formulation in the World Health Organization (WHO) and EMA guidelines, and they have been included in the Pediatric Investigation Plan (PIP) for the development of pediatric novel drug dosage forms [19,20,21,22]. For all patients struggling with swallowing difficulties, orodispersible minitablets (ODMTs) are particularly recommended as they eliminate the risk of choking. ODMTs, when placed in the mouth, quickly dissolve or disintegrate in the saliva (below 30 s) [20,23,24]. It has been proven that even 6-month-old infants can safely swallow a single minitablet in the orodispersible form [25,26,27]. Reviewing the literature reports, there is a growing tendency toward the design of minitablets (in orodispersible form as well), e.g., with carbamazepine, sildenafil, enalapril maleate, melatonin, rupatadine fumarate or hydrocortisone (utilizing various obtainment techniques), indicating the validity and demand of designing such a drug dosage form [24,28,29,30,31,32,33]. However, until now, ODMTs are still a new approach in pediatric drug delivery with a limited number of commercially available products.

Hydrocortisone (HT) is a glucocorticosteroid frequently used in children, primarily with adrenal dysfunction, as a replacement therapy. It is also administered in diseases of autoimmune etiology, severe and chronic allergic conditions, acute rheumatic myocarditis or ulcerative colitis and Crohn’s disease (in the acute phase) [34,35,36,37,38]. The usual recommended dose in children and adolescents is 0.4–0.8 mg/kg of body weight daily in two or three divided doses (Table S1) [38]. Significantly, HT is being administered in variable doses for one patient per day as the peak of endogenous cortisol secretion occurs early in the morning; therefore, the first dose to be administered upon awakening is the higher one, while the last, lower dose, is given 4 to 6 h before bedtime according to the chronopharmacology of glucocorticosteroids [34,35,36,37,38].

It has to be highlighted that doses of internationally registered conventional hydrocortisone tablets (10 mg or 20 mg), which are prescribed most often due to their widespread availability and relatively low price, are intended only for adults. When administering traditional tablets, dosing in younger children involves splitting the tablets, often into four parts or more, while in the case of newborns and infants, providing an adequate HT dose is impossible, thus, establishing the appropriate dosage regimen is troublesome, and pharmacy-compounded capsules often show non-compliance in mass and content uniformity [11,38,39,40,41,42]. On the global pharmaceutical market, there is one preparation registered for children: Alkindi^®^ Sprinkle (Eton Pharmaceuticals, Inc., Deer Park, TX, USA)—granules enclosed in a capsule (available doses: 0.5 mg, 1.0 mg. 2.0 mg, 5 mg), which are intended to be either poured directly onto the child’s tongue, or onto a spoon and placed in the child’s mouth [43]. Obviously, such a drug dosage form is much more child-friendly; however, it still requires manipulation and swallowing a certain amount of the granules, which can stick to the throat palate. The drug content in the ODMTs prepared in this study (single-dose form with 0.5 or 1.0 mg HT that disintegrates quickly in the mouth) can be freely adjustable by multiplication depending on age and weight (Table S1) and might be used as a unit of initial and increasing dosage. Compared to current age-appropriate formulations, ODMTs possess the potential to ensure precise, accurate and flexible dosing schedules for various age groups (including neonates and infants), simultaneously constituting a ready-to-use product with enhanced stability, improved palatability, and reduced potential of errors resulting from drug manipulation during administration.

The acceptable taste of drug dosage form is a significant factor when designing a formulation intended to be given to a child. It is also of high importance owing to the direct contact of the orodispersible preparation with the taste buds while dissolving in the mouth, as an unpleasant taste sensation carries the risk of patient nonadherence or even discontinuation of therapy. It is crucial to not underestimate that assessing the taste properties of the active substance at the very beginning of designing a drug formulation greatly facilitates research at later stages [44,45]. Among the numerous taste-masking methods utilized in pharmaceutical technology (e.g., flavors, sweeteners or ion exchange resins addition, granulation, microencapsulation), obtaining the solid dispersions of the drug with taste-masking polymers can be distinguished [46,47,48]. A variety of techniques ensure the preparation of solid dispersions, e.g., hot melt extrusion, spray drying, the solvent evaporation method or supercritical fluid precipitation [47,49,50,51,52]. Also, such dispersion may be obtained by milling the active pharmaceutical substance (API) with the inert polymer under specified conditions. The process results in a decrease in particle size, shape and surface structure of the API being dispersed molecularly or as ultrafine crystals in the matrix of an amorphous carrier (polymer), which limits the sense of the bitter taste by the formation of a physical barrier between the drug and the taste buds [47,53,54]. HPMC is one of the most widely used cellulose derivatives for creating polymeric dispersions. This water-soluble cellulose ether is resistant over a wide pH range and does not feature highly strong hydrogen bond donor and acceptor groups due to the lack of electrostatic charge. Therefore, HPMC proves a decreased risk of drug interactions, which makes the substance very relevant in pharmaceutical applications. Importantly, HPMC is classified as Generally Recognized As Safe (GRAS) and is approved to be used in pediatric drug dosage forms [55,56,57]. The most frequently used taste evaluation method is a simple trial with healthy volunteers; however, for safety and ethical reasons, taste evaluation in this way is difficult in pediatrics. Therefore, in vitro taste evaluation methods have been developed, which include the use of an electronic tongue (syn. artificial tongue, e-tongue) [58,59,60].

The purpose of this study was to develop 2 mm orodispersible ODMTs with HT in pediatric doses (0.5 mg—F1 and 1 mg—F2), with a disintegration time below 30 s, relevant pharmaceutical properties and acceptable taste, attained by preparing drug–polymer solid dispersion in a ball mill. Research focusing on the tableting of solid dispersion with HT as a taste-masking method to prepare ODMTs, to our best knowledge, has not been published so far. Moreover, there are no formulations available on the pharmaceutical market containing HT suitable for the pediatric population at required doses, considering acceptability, swallowability and dosing flexibility without the need to manipulate while being administered to a child. In order to investigate the taste-masking effectiveness, in vitro methods, including drug dissolution and an artificial “electronic tongue”, and in vivo tests, involving human taste panels, were used. To provide deeper insight into the character of HT and excipients, interactions between components and the influence of utilized technology of obtaining ODMTs (differential scanning calorimetry (DSC) and Fourier-transform infrared spectroscopy (FTIR)) were analyzed. Also, considering that the objective of the research was to design an innovative drug dosage form, preliminary bioequivalence studies of prepared ODMTs with reference products available internationally (Alkindi^®^ Sprinkle (Eton Pharmaceuticals, Inc., Deer Park, TX, USA)—granules enclosed in capsules, and Hydrocortisone SF^®^ (Sun-Farm Sp. z o.o., Łomianki, Poland)—conventional tablets [61]) were conducted utilizing an animal model.

## 2. Materials and Methods

### 2.1. Materials

HT was purchased from Fagron, Modlniczka, Poland. Prosolv^®^ ODT was purchased from JRS Pharma, Patterson, NY, USA. Hypromellose (Pharmacoat^®^ 605) was obtained from Shin-Etsu Chemical Co., Ltd., Tokyo, Japan. Magnesium stearate was acquired from POCh Piekary Śląskie, Poland.

### 2.2. Preparation of Tableting Blends and ODMTs

The compositions of the formulated blends are presented in Table 1. Five types of ODMTs (F1-F5) were obtained.

#### 2.2.1. Ball Milling Process

To prepare formulations F1 and F2, a ball mill grinder was utilized (Retsch MM 400, Verder Scientific, Haan, Germany) at a setting of 20 min, 15 Hz. Mixtures of HT and HPMC in a 1:1 ratio were initially mixed in InvoMatic (FagronLab, Modlniczka, Poland) and then transferred to steel jars containing 2 zirconia spheres with 7 mm diameter. The milling parameters were selected by earlier preliminary tests, and the process was carried out at room temperature. Formulations F3, F4 and F5 served to compare the effectiveness of bitterness reduction in F1 and F2; therefore, the ball milling stage was eliminated during their preparation.

#### 2.2.2. Granulation Process

In all prepared blends (F1-F4), the compression was preceded by granulation of the powder mixture with 5% N-vinylpyrrolidone (NVP) ethanolic solution. Obtained granulates were dried at room temperature and mixed automatically with the other excipients (InvoMatic, FagronLab, Rotterdam, The Netherlands) to obtain fine particles.

#### 2.2.3. Compression Process

ODMTs were prepared via a compression method using a traditional single-punch tablet machine (Type XP1, Korsch, Berlin, Germany) equipped with 2 mm diameter punches. In order to set the appropriate parameters of the direct compression, different pressure force values ranging from 0.4 to 1.4 kN were tested. It was determined that tablets with optimal properties (i.e., maintained hardness and had a short disintegration time) were obtained using a 0.7 kN force.

### 2.3. Method Validation

The procedure involved the determination of the most important parameters subjected to the validation process such as linearity, accuracy, intra-day and inter-day precision, limit of determination (LOD) and limit of quantification (LOQ). The calibration curve was obtained by performing a series of HT dilutions in phase (to establish drug content), saliva-imitating fluid (for in vitro HT release) and a rat blood plasma (for bioequivalence studies). The accuracy and precision of the method were estimated based on the results of 9 parallel determinations for three different analyte concentration levels (3 determinations at each level of the content of the determined component in the sample) determining the recovery of the analyte, RSD and %CV parameters. In order to find the range of linearity of the calibration curve, 3 series of standard solutions were prepared at 5 concentration levels of the analyte. Calibration curves were made in the concentration range covering the expected content of hydrocortisone in samples, and the LOQ was 10 ng/mL. The smallest concentration of a determinable component (LOD) in the tested sample, that can be detected by a given method with a specified probability, was determined as 3.28 ng/mL. The validation parameters are shown in Table 2.

### 2.4. Quality Assessment of Tableting Blends and ODMTs

The properties of formulated ODMTs were evaluated according to the European Pharmacopoeia 11.0 (Ph. Eur.) [62]. Each test was for 20 randomly selected ODMTs.

#### 2.4.1. Flow Properties of Tableting Blends

To assess the compressibility of the powders, the apparatus Electrolab ETD-1020 (Mumbai, India) was used. The bulk and tapped densities were verified, and the powder density index (Carr Index) and powder flow index (Hausner’s ratio) were computed.

#### 2.4.2. Uniformity of Weight and Thickness

ODMTs were weighted individually employing analytical balance (Radwag, Radom, Poland). The thickness was measured using a calibrated digital caliper (Beta1651DGT, Milan, Italy).

#### 2.4.3. Mechanical Properties

The hardness of the tablets, expressed as breaking force, was analyzed using a hardness tester (5Y, Pharmaton AG, Thun, Switzerland). Friability was determined using a Friabilator (USP) tester EF-1 W (Electrolab, Mumbai, India) according to a pharmacopoeial monograph (6.5 g of dedusted minitablets were utilized) [62]. ODMTs were reweighted, and the mass was compared with their initial weight (automatically by Friabilator tester, expressed as % of weight loss).

$$\mathrm{Friability}\;(\%)=\frac{W1-W2}{W1}*100$$

W1 = initial weight of ODMTs before the friability test; W2 = weight of ODMTs after the friability test.

#### 2.4.4. Drug Content

HT content uniformity for individual ODMT was performed by HPLC Agilent Technologies 1200 (Agilent, Santa Clara, CA, USA) using Zorbax Eclipse XDB-C18 5 μm 4.6 × 150 mm column (Agilent, USA). ACN:MeOH:H2O (25:25:50, *v*/*v*) was used as a mobile phase (modified according to Ph. Eur. [62]) with a flow rate set at 1.0 mL/min and a wavelength of 254 nm. The standard calibration curve was linear in the range of 5–100 µg/mL, and the correlation coefficient R^2^ equaled 0.999 (point 2.3., Table 2).

#### 2.4.5. Disintegration Time Assessment

##### Human Taste Panel

The study was carried out in vivo by eight healthy volunteers (Research Ethics Committee at the Medical University of Białystok, approval number APK.002.313.2023) in accordance with the given scheme: rinsing the mouth, placing a single ODMT on the tongue until disintegration, and spitting out. The time required for the total disintegration in the oral cavity was observed and measured with a stopwatch.

##### Petri Dish

A Petri dish (7 cm diameter) was filled with 4 mL of saliva-imitating fluid (phosphate buffer pH = 6.8, corresponding to saliva pH, composed of Na_2_HPO_4_; KH_2_PO_4_ and water; adjusted to pH 6.8 by 1M NaOH) [63,64], and a single ODMT was positioned in the middle. The time of total ODMT disintegration into fine particles was determined.

#### 2.4.6. Differential Scanning Calorimetry (DSC)

A thermal analysis of the HT was performed using a thermal analyzer system (DSC Mettler Toledo, Greifensee, Switzerland). The following samples: pure HT, pure HPMC, their physical mixture and the solid dispersion obtained after milling, were accurately weighed in an aluminum pan and tested. Thermograms were taken within the temperature range of 40 °C to 300 °C, with a heating rate of 10 °C per minute and a continuous flow of nitrogen flow of 20 mL/min. The samples were encapsulated in metal (aluminum) pans. The pan without any content served as the reference.

#### 2.4.7. FTIR Analysis

Attenuated Total Reflection Fourier Transform Infrared Spectroscopy (ATR-FTIR) was applied to determine the chemical characteristics of utilized substances, granulated HT:HPMC 1:1, milled HT:HPMC 1:1 and ODMTs. The study was carried out using a ThermoScientific Nicolet 6700 FTIR spectrometer (Thermo Scientific, Dreieich, Germany) equipped with a DLaTGS detector and KBr beam splitter, in the scan range from 500 to 4000 cm^−1^ at the resolution of 4 cm^−1^ and with a scan number of 32.

### 2.5. Evaluation of Taste-Masking Effectiveness

#### 2.5.1. In Vitro HT Release

In vitro release of HT from prepared ODMTs was performed in a paddle apparatus (Erweka Dissolution Tester DT 600HH, Heusenstamm, Germany) using 100 mL of saliva-imitating fluid (phosphate buffer pH = 6.8, corresponding to saliva pH, composed of Na_2_HPO_4_; KH_2_PO_4_ and water; adjusted to a pH of 6.8 by 1M NaOH) [63,64] with the addition of Tween 80 in 1% concentration. The apparatus was constantly rotated at 75 rpm, and the bath temperature was 37 °C (+/−0.5). The amount of released HT was assessed using the HPLC method, as described in Section. 2.4.4. The method validation parameters are shown in Section 2.3., Table 2.

#### 2.5.2. In Vivo Evaluation with Healthy Volunteers

This research was performed according to the Declaration of Helsinki, and the protocol was authorized by the Research Ethics Committee at the Medical University of Bialystok (approval number APK.002.313.2023). The effectiveness of the taste-masking level was carried out by eight healthy volunteers as follows: ODMTs were placed on the tongue for 30 s (the maximum time to dissolve/disintegrate according to FDA guidelines), spit out, and then their mouths were rinsed with water. A sensory assessment was determined using the following point scale: 0—no bitterness, 1—slightly bitterness, 2—moderately bitterness, 3—significant bitterness. Prior to the test, a preliminary selection of participants was carried out by performing a threshold assessment test for individual basic tastes, using solutions of standard substances: sour—tartaric acid, sweet—sucrose, salty—sodium chloride, bitter—quinine. Additionally, each participant of the study determined whether the preparation was acceptable in terms of visual assessment, smell, taste and texture by awarding points: “yes” answer—1, “no” answer—0. Preparations that achieve ≥ 50% of the points were considered acceptable.

#### 2.5.3. Electronic Tongue—Sensor Array Fabrication and ODMTs Measurements

A laboratory version of a potentiometric electronic tongue was used for the evaluation of taste-masking efficiency [60]. A sensor array was composed of 8 types of solid-state ion-selective electrodes (ISE), equipped with chemosensitive membranes, the compositions of which are listed in Table 3. Two electrodes were prepared for each membrane type.

The procedure applied for electronic tongue analysis is a standard measurement protocol that has been applied previously for various pharmaceutical samples to study taste-masking efficiency [60,65,66]. It was based on the following steps: signal stabilization for 5 min (sensors immersed in deionized water), the introduction of the studied pharmaceutical formulation to the medium and recording of electrode signals that are influenced by the released active pharmaceutical ingredient (API) and excipients, in time. The signals of the sensors were registered for 10 min (5 min stabilization, 5 min release). Between sample measurements, the ISEs were washed with purified water and dried. The resulting signals were processed using Principal Component Analysis (PCA) performed in SOLO^®^ version 8.9 software (Eigenvector Research Inc., Wenatchee, WA, USA).

### 2.6. Bioequivalence Studies

The determination of HT profiles in animal models for F1 and F2 in comparison with commercially available products (Alkindi^®^ Sprinkle 1 mg capsules—reference A, Hydrocortisone SF^®^ 10 mg tablets—reference B) was conducted according to the scheme presented in Table 4. Each study group (I-IV) was divided into 5 subgroups (A, B, C, D, E,) with 6 rats in each one. The number of subgroups corresponded to the 5 time points scheduled for sampling (A—0.5 h; B—1 h; C—2 h; D—4 h; E—8 h). Each rat in the study groups I and II received ODMTs orally according to an established regimen (Table 4) via an intragastric probe. For groups III and IV, the equivalent of 1 mg HT (the granules of Alkindi^®^ Sprinkle or powdered Hydrocortisonum^®^ SF tablet) was suspended in a 2% hydroxyethylcellulose (HEC) solution and administered using a suitable oral probe. The volume of the carrier did not exceed 1 mL/kg [67,68,69]. Before each procedure, rats were sedated using isoflurane and kept under constant observation on a heating mat with monitored body temperature. The depth of anesthesia was controlled and maintained with 1.5–2% of isoflurane solution.

To establish the bioequivalence of the tested formulations with reference products, the following pharmacokinetic parameters were determined: the maximum (peak) serum concentration (C_max_), the time to maximum plasma concentration (T_max_), the half-life (T_0,5_)_,_ the area under the curve (AUC) from zero to the last sampling point (8 h—AUC_0–8_) and up to infinity (AUC_0–∞_) and also extent of bioavailability (EBA). C_max_ was obtained directly from the dependence of concentration–time curve data as the maximum observed concentration, while T_max_ was the time at which C_max_ was noted. The linear trapezoidal method was used to calculate the area under the serum concentration–time curve. The terminal elimination rate constant (λz) was estimated at the terminal phase by linear regression after log-transformation of the concentrations using at least 2 points, and t_0,5_ was calculated as ln2/λ_z_. The EBA parameter was estimated as follows:


$$EBA=\frac{Ds*{AUC}_{X}}{Dx*{AUC}_{s}}*100$$

EBA—extent of bioavailability; D_x_—dose of the tested drug; D_s_—dose of the reference drug; AUC_x_—area under the curve of the tested drug; AUC_s_—area under the curve of the reference drug.

The non-compartmental method was used for the determination of pharmacokinetic parameters in bioequivalence studies.

#### 2.6.1. Sampling to Evaluate HT Concentration; HPLC Analysis

In order to estimate HT concentrations, the plasma samples were deproteinized with methanol (MeOH), mixed (Vortex, DanLab, Warsaw, Poland), and centrifuged at 15,000 rpm for 20 min at 4 °C. The supernatant fluid was passed through 0.22 µm filters, and 20 µL was injected into the HPLC system for analysis. The concentration of HT in the samples was determined using the HPLC method according to Section 2.4.4. The analytical method was validated according to the criteria used for the analysis of biological samples. The method validation parameters are shown in Section 2.3., Table 2.

#### 2.6.2. Statistical Analysis

The normally distributed data were studied using a one-way analysis of variance (ANOVA) or unpaired Student *t*-test and shown as mean ± SD. The statistical analyses were performed using GraphPad PRISM 9.0 software. The differences were deemed statistically significant when *p* < 0.05.

## 3. Results and Discussion

### 3.1. Pharmaceutical Evaluation of Obtained ODMTs

A ready-to-use commercial mixture—Prosolv^®^ ODT (containing microcrystalline cellulose, colloidal silicon dioxide, mannitol, fructose and crospovidone) was used for obtaining ODMTs. As the mixture is manufactured by the co-drying process [70], it results in material characterized by optimized properties in terms of particle size, shape and porosity, compared to the physical mixture of the individual components; therefore, such a mixture is very useful in the tableting process [70,71]. Moreover, each of the ingredients is classified as GRAS and tolerated by children according to the European Paediatric Formulation Initiative (EuPFI) in the Safety and Toxicity of Excipients for Paediatrics (STEP) database [22,72,73].

During the tableting process, the crucial parameter affecting the uniform matrix filling is the flowability of the tablet mass. It is particularly important in the case of small-diameter tablets, and it affects obtaining a formulation with uniform weight and drug substance content [74]. Therefore, it is necessary to thoroughly consider and select the composition and technology while preparing the tableting blends. Obtaining ODMTs by simply mixing HT with Prosolv^®^ ODT and magnesium stearate prior to the direct compression posed significant technological difficulty and proved to be impossible (despite the presence of excipients with antiadhesive properties such as colloidal silicon dioxide, magnesium stearate and combining the components using a mechanical grinder), as HT adhered to the punches extremely well. Therefore, HT along with HPMC were granulated utilizing 5% ethanolic solution of NVP. Prepared, dried granulates, with the humidity not exceeding 5%, along with Prosolv^®^ ODT and magnesium stearate were mixed in InvoMatic (FagronLab, Modlniczka, Poland) to obtain a fine powder. The granulation step significantly improved the tableting process. As an attempt to mask the bitter taste of HT, HT and HPMC (1:1) were ground in a ball mill (for F1, F2) to obtain solid dispersion. The mixture obtained by grinding in the ball mill also could not be introduced into the tableting mass due to poor flowability, as well as sticking and picking phenomena. Therefore, it was subjected to granulation as above. The composition and technology for obtaining the individual tableting blends are presented in Table 1, Section 2.2. The tablet masses were subjected to a preliminary quality assessment (powder flowability) in accordance with Ph. Eur. requirements [62]. The measurement of the angle of repose was used as the primary test of the flowability of the tablet masses. In addition, the Hausner ratio and density index (Carr index) were calculated. Obtained results indicated that the grinding process in a mechanical mill of the previously prepared granulates significantly improved the flowability of the tablet masses (Table 5).

It should be highlighted that the difficulty in designing orally disintegrating tablets is the simultaneous maintenance of a short disintegration time and optimal parameters such as friability and mechanical strength. Orodispersible tablets are usually produced with low compression force, which requires optimization of composition (appropriate particle size, compressibility, bulk density). During their manufacture in the tableting process, the key parameter is the choice of the compression force applied, whereby the use of too low a force can lead to a very brittle product, while the application of a higher compression force leads to obtaining a robust form but at the expense of increased disintegration time. To determine the appropriate conditions of the tableting process, different force values were tested in the range of 0.4 to 1.4 kN. It was observed that below 0.4 kN, the tablets could not be formed as they were too brittle, while above 1.4 kN, those obtained were characterized by hardness, resulting in an unsuitable disintegration time of more than 30 s, which was an undesirable effect for orally disintegrating tablets. ODMTs with satisfactory parameters (with simultaneously preserved mechanical properties and disintegration time of less than 30 s) [75] were maintained using a pressing force of 0.7 kN. Tests of friability and hardness proved that obtained ODMTs were characterized by good mechanical properties. The hardness of tablets prepared with a higher dose of HT (F2) was lower compared to F1. The friability for both F1 and F2 was below 1%, meeting the pharmacopoeial requirements (a maximum weight loss of not more than 1% is considered acceptable). As the Ph. Eur. does not provide a monograph for minitablets, the studies were based on the information for conventional, uncoated tablets with an unmodified release profile [62]. For the quality tests, only F1 and F2 were employed since they represent the intended, final product. The specifications of the obtained ODMTs are shown in Table 6.

The Ph. Eur. defines orally disintegrating tablets as uncoated tablets intended to be placed in the mouth, where they rapidly disintegrate before being swallowed within 3 min [62]. However, according to FDA guidelines, the need to reduce the disintegration time of these tablets to 30 s or even below has been indicated [75]. Such a disintegration time is therefore being pursued in pre-formulation studies. Disintegration time tests were conducted involving two methods: in vivo by a human taste panel and under conditions imitating those prevailing in the oral cavity utilizing a Petri dish with 4 mL of the artificial saliva (see Section 2.4.5.). Regardless of the method, the disintegration time of designed ODMTs (F1–F5) was below 30 s.

**Figure 1:**
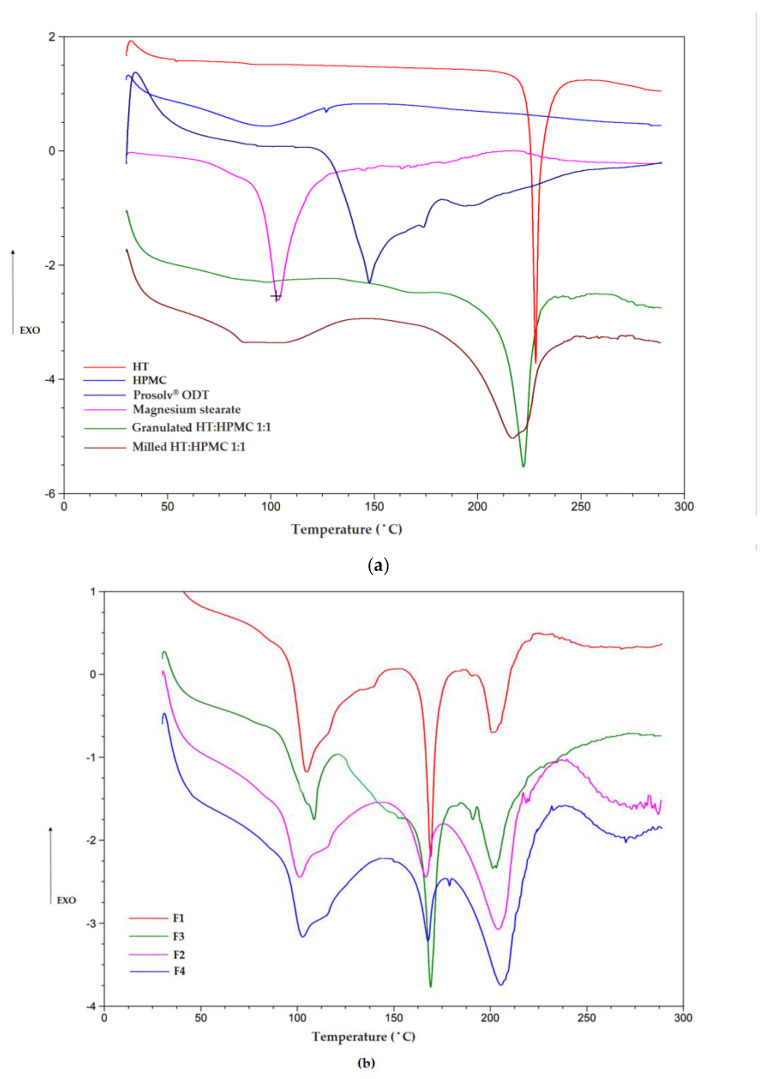
DSC thermograms of (**a**)—HT, HPMC, Prosolv^®^ ODT, magnesium stearate, granulated HT:HPMC 1:1, milled HT:HPMC 1:1 and (**b**)—designed ODMTs: F1, F2, F3, F4.

**Figure 2:**
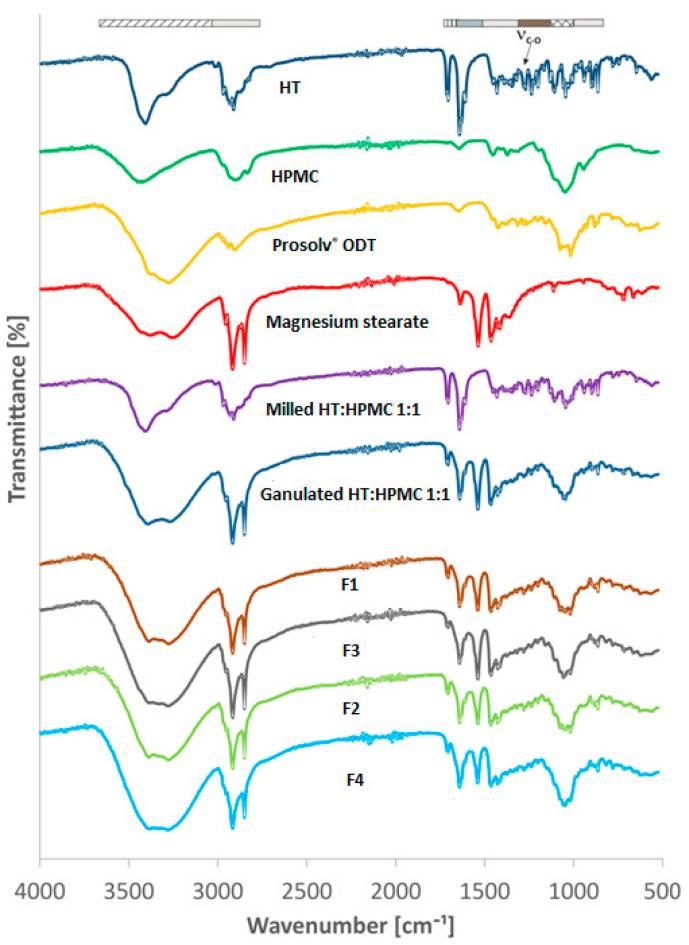
FTIR analysis of HT, HPMC, Prosolv^®^ ODT, magnesium stearate, milled HT:HPMC 1:1, granulated HT:HPMC 1:1, F1, F3, F2, F4.

An appropriate selection of pharmaceutical excipients and manufacturing technologies is a key issue while creating drug dosage forms. Differential scanning calorimetry (DSC) is one of the analytical techniques frequently applied to determine physical drug properties, as well as to investigate potential incompatibilities with other components. The procedure provides detailed information about the presence of energetic properties of substances, pointing to the differences in the heat flow generated or absorbed by the sample. This is particularly important in the case of formulations subjected to grinding in a ball mill, where the generated energy can affect the drug conformation. According to the literature data, the HT melting point oscillates around 220 °C [76,77]. As depicted in Figure 1a, the thermogram reveals a sharp, distinct endothermic peak of pure HT at 227.75 °C, highlighting a definable and tight temperature phase transition range, with a distinct melting point to crystalline API. In addition, an assumed sample decomposition after melting was observed—the exothermic event transition is shown at 241.35 °C. No additional thermal events connected with decomposition or loss of surface water were observed. The thermogram of raw HPMC showed a broad endothermic transition commencing at approximately 75 to 120 °C with a peak value of 96.19 °C. Granulate composed of HT and HPMC in a 1:1 ratio exhibited a sharp peak corresponding to HT at 221.34 °C and for ball milled formulation at 216.61 °C. The peaks have been slightly shifted towards lower temperatures. This is probably due to the fact that both granulation and milling involve the application of mechanical energy to physically break down coarse particles into finer ones which can affect the structure of the active substance. During the ball milling process, the high rotation speed and therefore collision between the balls (which improves the grinding performance) in a container generates high pressure and energy and the obtained solid dispersion is a physical mixture with highly dispersed drug crystals in the carrier. There was a reduced intensity in granulated and milled formulations; nevertheless, endotherms confirming HT crystallinity were displayed. In Prosolv^®^ ODT, the principal component is mannitol, with melting points from 155 °C to 165 °C [78]. The peak corresponding to magnesium stearate was observed at 103.22 °C [79]. Characteristic peaks of mentioned ingredients were noted in each of the obtained ODMTs (F1-F4). However, some shifts in the location of HT melting peaks were observed in the designed formulations (Figure 1b). A possible explanation for a such result is partly dissolution of HT (present in milled or granulated form) at the mannitol melting point HCT, high miscibility with the components and also partial binding of water to the structure, which formed intermolecular forces that affected the existing endothermic processes. Overall, the obtained results suggest that interactions between the components did not occur, and the techniques introduced in ODMT preparation did not affect the final drug dosage form, which has been confirmed by FTIR spectroscopy (Figure 2).

DSC was supported by another non-thermal technique—FTIR—to determine if any intermolecular interactions or chemical reactions might have occurred between HT, HPMC, the physical mixtures and co-processed excipients during the milling and compression process or if the process did not affect the physicochemical nature of HT. The IR spectra are presented in Figure 2. The pure HT possesses characteristic adsorption bands at 3406.03 cm^−1^, 2926.16 cm^−1^ for O-H and C-H stretching vibration, respectively. The band for C=O stretching vibration occurs at 1713.6 cm^−1^. The band at 1629.48 cm^−1^ is due to aromatic C=C stretching vibration. The band at 1269 cm^−1^ is the result of C-O stretching vibration [80]. A detailed inspection of the FTIR data for mixtures revealed that characteristic absorption bands of HT were found in all the spectra. Furthermore, the absence of a shift in the characteristic absorption bands of the components, combined with the absence of new bands, implies that no physical interaction or chemical reaction occurred between the components. This is consistent with the results obtained in the DSC analysis and indicates that HT was stable in the prepared formulation and did not change during processing. Drug crystallinity was maintained after the milling, granulating and tableting processes.

### 3.2. Taste-Masking Assessment

Due to the direct contact of ODMTs with the taste buds, eliminating the bitter taste of the active substance is an important factor affecting the effectiveness of the pharmacotherapy and determining the patient’s adherence. The evaluation of taste-masking effectiveness of the designed ODMTs was carried out utilizing three independent methods: based on the HT release study, sensory evaluation with the participation of healthy volunteers, and using a chemical taste detector—“electronic tongue”.

#### 3.2.1. In Vitro Drug Release

**Figure 3:**
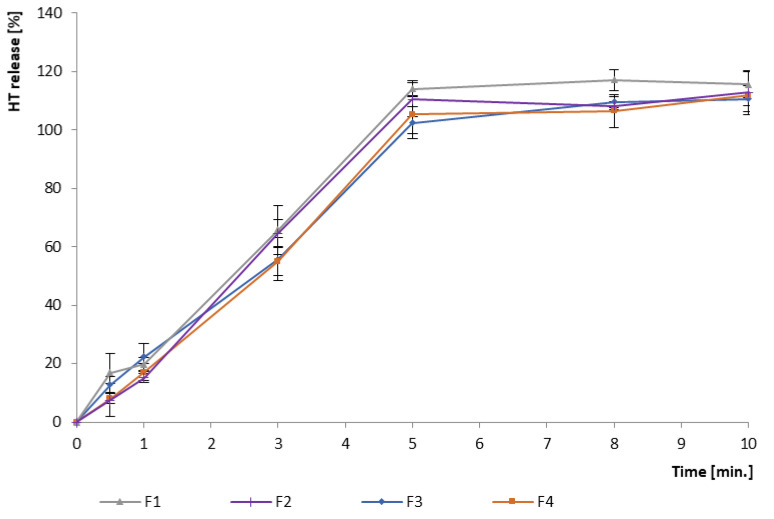
HT release profile in pH 6.8 phosphate buffer.

As exhibited in Figure 3, just a slight fraction of the dose was released up to 30 s (the time when the ODMT disintegrates)—a maximum of about 18%, and HT release profiles from all formulations were comparable. Also, up to 1 min, the increase in the released dose was not observed. The content of HPMC in a single ODMT is minor—about 7% of the mass in F3 and F1 and 15% in F2 and F4; therefore, it did not influence the HT release profile, but provided taste masking while in the mouth. Additionally, the formation of solid dispersion did not lead to a change in the dissolution profile. The obtained results indicate a satisfactory taste masking effect considering a very quick disintegration time (below 30 s) and short residence time in the oral cavity.

#### 3.2.2. In Vivo Human Taste Panel

**Figure 5:**
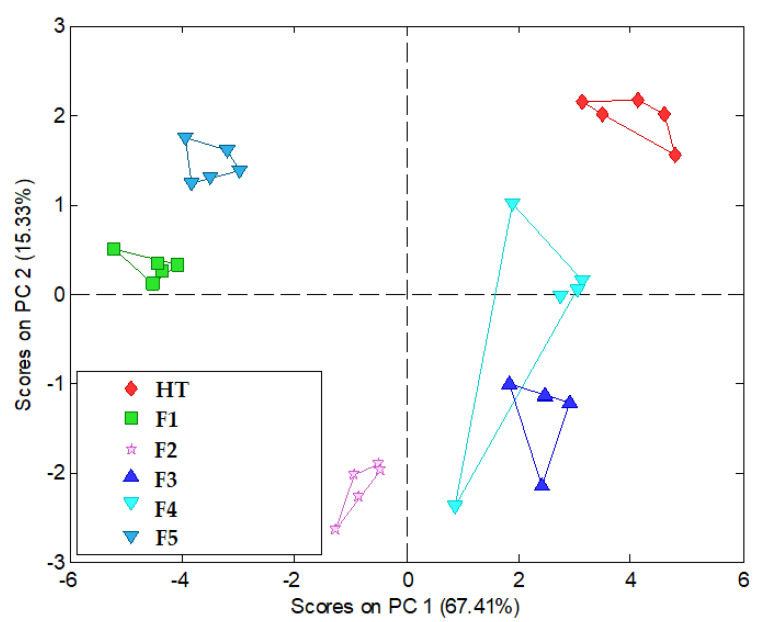
PCA score plot of electronic tongue results.

The number of ODMTs used for taste evaluation in the human test panel was determined on the basis of the developed HT dosing scheme in the pediatric population (Table S1) in accordance with the doses usually used regarding clinical data [38]. It was assumed that the dose of HT to be administered to a participant for a single dose is 2.5 mg (5 units of F1, 3 units of F2). In terms of acceptability, each formulation was considered as acceptable. Regarding appearance, smell and texture, all participants awarded ODMTs (all formulations) 1 point each (see point 2.4.2.). However, the differentiating aspect was taste—in these terms, the formulation determined as the best with regard to the elimination of the bitter taste sensation was F1 (Table 7). Certainly, the taste-masking effect was partly influenced by the presence of mannitol and fructose in the ready-to-use mixture—Prosolv^®^ ODT. It also should be noted that the presence of mannitol in the formulation, apart from providing sweetness, causes the feeling of a cooling effect when dissolving in the mouth which positively affects the taste sensation. Worth mentioning is that the sweetness in the oral formulation is preferred by the patients (sweetened pharmacy-compounded powder containing HT and tasteless dry HT granules were compared) [37]. However, according to the obtained results, it was not enough to mask the taste appropriately (Table 7, Figure 5). Simply mixing with sweeteners did not result in significant improvement as the drug molecule was unbound to sweetener molecules, and therefore, the tongue distinguished two of them separately. The best taste-masking level was achieved for ODMTs containing solid dispersion in their composition (F1, F2). The presence of solid dispersion, where HT was being dispersed in the polymer, created a physical barrier between the drug and the taste buds, thus affecting the taste sensations.

#### 3.2.3. In Vitro Electronic Tongue Utilization

**Figure 4:**
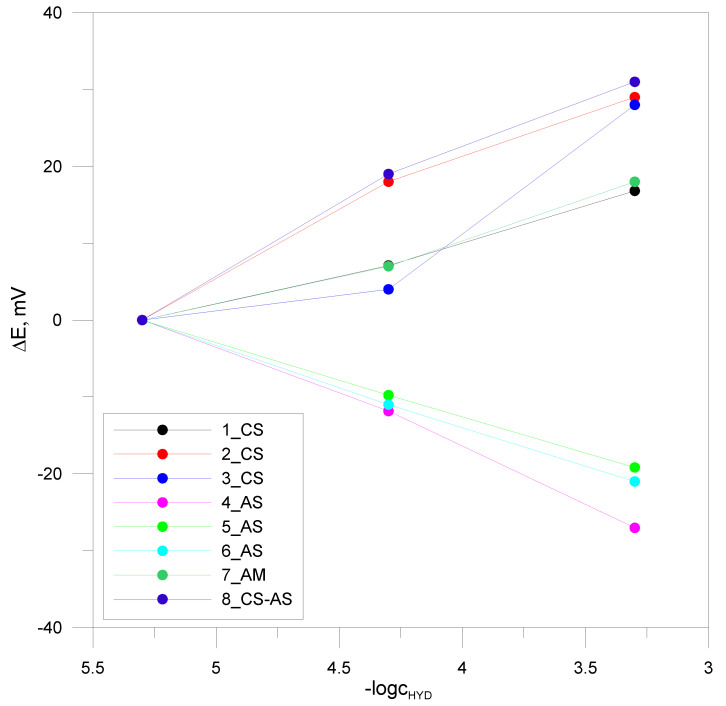
Potentiometric response of the selected electrodes with a cationic and anionic function.

A further method to test the effectiveness of taste-masking efficiency in obtained ODMTs was the utilization of a custom-designed electronic tongue. To ensure appropriate performance of the device, all sensors were tested towards pure API—HT. The signals of electrodes immersed in solutions of HT of decreasing concentration from 5·10^−4^ to 5·10^−6^ M were recorded, and the respective calibration curves are presented in Figure 4.

The obtained results indicate the differing responses of the electrodes. The cation-selective, amine and cation–anion electrodes exhibited an increasing function with increasing concentration, whereas the electrodes containing TDMAC and TDAC demonstrated an anionic function. All electrodes exhibited lower than Nernstian sensitivity for HT ranging from about 10 to 15 mV/decade. This may be due to the structure of the HT molecule, which lacks typical ionic groups. The linearity range of the characteristics is from 5·10^−4^ to 5·10^−6^ M with good correlation coefficients of approximately 0.988 to 0.999. However, for sensor 3_SC, this range was limited to 5·10^−4^ to 5·10^−5^ M, while for sensor 6_AS, it was between 5·10^−5^ and 5·10^−6^ M. The parameters of the electrodes, particularly their varied sensitivity, were suitable for creating a sensor array. The prepared sensor array of the electronic tongue was applied to check the taste-masking efficiency in the studied formulations—ODMTs. Such a test relies on the comparison of the developed formulations with placebo and pure API, which serves as a bitterness standard. Thus, signals of the ISEs were recorded according to the procedure described in the experimental section for six types of samples: four types of ODMTs (F1–F4), F5 (placebo), and pure HT. The electronic tongue results in the form of a PCA score plot are presented in Figure 5.

All samples formed easily separable, distinct clusters. Chemical images of placebo and API were placed on opposite sides of the PCA score plot and were characterized by an opposite PC1 value (PC—principal component), carrying most of the variability of the studied dataset (almost 70%). Almost all formulations (F2, F3, F4) were placed between them in terms of PC1 value, which suggests an intermediate bitter taste. Only F1 had a very similar PC1 value to the placebo and was the closest to the placebo cluster, which can be related to the best taste-masking effect. It correlated well with the sensory assessment (Table 7), in which this formulation gained the lowest scores of bitterness; moreover, it was indiscernible from the placebo for three volunteers. The electronic tongue results suggested that a lower taste-masking effect was observed for F3 and F4 because their chemical images were close to the chemical images of HT. They were also the only formulations that had positive PC1 values, observed also for pure API. Emphasizing, it correlated with the sensory assessment, where the two ODMTs were assessed as moderately/very bitter. F2 was characterized by the negative value of PC1 but was placed at a distance from the placebo cluster. Thus, in terms of the PC1 value, the following sequence of bitterness degree was estimated: F5 (placebo, lowest bitterness) < F1 < F2 < F3 < F4 < HT (highest bitterness). The electronic tongue results were perfectly consistent with those of the sensory assessment presented in Table 7.

### 3.3. Bioequivalence Studies

**Figure 6:**
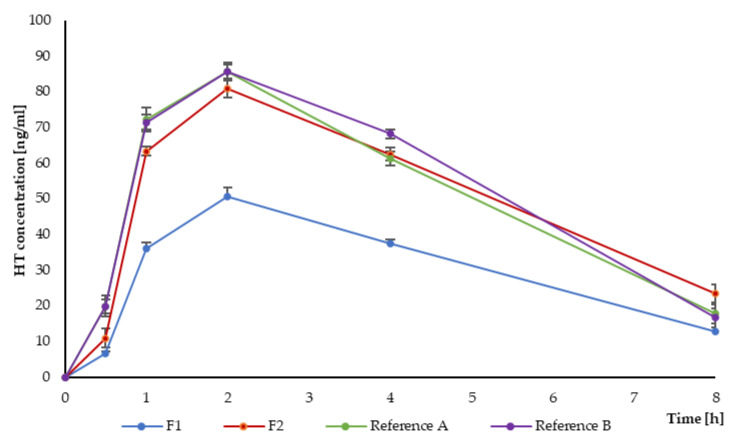
Mean HT concentration (n = 6) after oral administration of F1 and F2 versus reference products A and B.

During the tableting process (compression force) and the solid dispersion preparation (milling), some changes in the crystallographic arrangement of HT might occur. Therefore, HT concentration was measured in the preliminary bioequivalence studies in animal models after drug oral administration (Figure 6).

The bioavailability parameters of the tested formulations and reference products are shown in Table 8 (see also Tables S2 and S3). In all tested formulations, T_max_ was 2 h, C_max_ was determined at 50.6 ng/mL and 81 ng/mL for F1 and F2, respectively, compared to 85.6 ng/mL and 85.7 ng/mL for reference products A and B. The AUC_(0–8)_ was determined for the F1 and F2, which was 245 ng × h/mL and 409 ng × h/mL, respectively, while for the tested reference products A and B, it was 413 ng × h/mL and 430 ng × h/mL, respectively. In order to compare the tested formulations with reference drugs, relative bioavailability was determined as the EBA of the drug. The formulations proved to be bioequivalent, considering that the EBA for reference product A and F2 formulations was 99%, and for reference product B, it was 95%. The sampling schedule covered the plasma concentration–time curve providing an estimate of the extent of exposure. According to European Medicines Agency (EMA) guidelines, it is achieved if AUC_(0-t)_ covers at least 80% of AUC_(0–∞)_ [81]. Drugs at a 1.0 mg dose were used as reference products. F1 (with 0.5 mg HT) exhibited AUC_(0–8)_ = 245 ng × h/m (Figure 6), which was about half the value of the reference products (EBA_Ref.A_ 59%; EBA_Ref.B_ 57%). This was consistent with the presented results, and it might be concluded that F1 proved bioequivalence with reference products. According to the performed ANOVA test (F-test(3.20) = 121.1), there was no significant difference in AUC between F2, reference A (*p* = 0.0503) and reference B (*p* = 0.1295).

## 4. Conclusions

The experiments were focused on the evaluation of HT taste-masking effectiveness by forming solid dispersions using ball mill grinding to obtain 2 mm ODMTs. The HT tableting process was preceded by obtaining a solid dispersion of hydrocortisone with a taste-masking polymer (hypromellose), and then the mixture was granulated (to improve the flowability of the tableting blend) with the wetting agent (5% NVP). The obtained granules were dried, homogenized, and after mixing with ready-to-use mixture—Prosolv^®^ ODT—subjected to the tableting process. Obtained ODMTs exhibited acceptable physical parameters, including a disintegrating time below 30 s, optimal hardness and friability. Formulations containing solid dispersion of HT and HPMC featured the best degree of taste masking. The results demonstrated the usefulness of the designed solid dispersion in masking the unpleasant taste of the active substance, and the most satisfactory taste-masking effect was indicated for formulation F1. Applying DSC and FTIR methods confirmed that the technology and excipients used to obtain ODMTs did not affect the pharmacological properties of the drug. Evaluation of the preliminary bioequivalence studies proved the bioequivalence of HT in comparison to immediate-release hydrocortisone granules and tablets. The designed formulations are featured with child-applicability potential and might constitute a suitable drug dosage form for delivering HT to children of all ages, which is so far lacking in the pharmaceutical market.

## Figures and Tables

**Table 1:** Composition of the tableting blends.

**Composition [%]**					
Formulation	F1 (0.5 mg)	F2(1.0 mg)	F3 (0.5 mg)	F4 (1.0 mg)	F5
	Ball-Milled, Granulated		Granulated		Placebo
**Ingredients**					
HT:HPMC 1:1	15	30	15	30	-
Prosolv ODT^®^	80	65	80	65	95
Magnesium stearate	5				

**Table 2:** Method validation parameters.

Method Validation Parameters	HT/Salvia Imitating Fluid	HT/Phase	HT/Blood Rat Plasma
Range	5–100 µg/mL	5–100 µg/mL	10–200 ng/mL
Linearity	*p* = 34.13C + 20.91	*p* = 40.29C − 33.62	*p* = 33.51C + 0.61
R^2^	0.999	0.999	0.999
Intra-day precision (*n* = 9)			
RSD	0.03	0.04	0.04
CV %	2.67	4.48	3.96
Accuracy			
Recovery %	97.49–99.57	98.27–102.04	95.33–99.94
Inter-day precision (*n* = 18)			
RSD	0.06	0.03	0.04
CV %	5.76	3.45	4.39
Accuracy			
Recovery %	98.14–103.96	95.33–99.94	99.86–101.10

**Table 3:** Sensor array of the applied electronic tongue system.

No	ISE ID*	Polymer	Plasticizer *	Lipophilic salt	Ionophore
1	CS	PVC, 66 mg	o-NPOE, 132 mg	KTFPB, 2.0 mg	-
2	CS		DOS, 132 mg	KTpClPB, 2.0 mg	-
3	CS		o-NPOE, 132 mg	KTpClPB, 2.0 mg	-
4	AS	PVC, 65 mg	o-NPOE, 128 mg	TDMAC, 7.0 mg	-
5	AS		DOS, 128 mg	TDMAC, 7.0 mg	-
6	AS		o-NPOE, 128 mg	TDAC, 7.0 mg	-
7	AM	PVC, 54 mg	DOS, 136 mg	-	Amine ionophore I, 10 mg
8	CS-AS	PVC, 64 mg	o-NPOE, 130 mg	TDMA-TCPB, 6 mg	-

* CS—cation selective; AS—anion-selective; AM—amine-sensitive; o-NPOE—2-nitrophenyl octyl ether; DOS—Bis(2-ethylhexyl) sebacate; KTFPB—Potassium tetrakis [3,5-bis(trifluoromethyl)phenyl]borate; KTpClPB—Potassium tetrakis(4-chlorophenyl)borate; TDMAC—Tridodecylmethylammonium chloride; TDAC—Tetradodecylammonium chloride; TDMA-TCPB—Tetradodecylammonium tetrakis(4-chlorophenyl) borate; PVC—poly(vinylchloride.

**Table 4:** Control and test groups of rats in bioequivalence studies.

Group Number	Group Description	Group Size (Number of Rats)		Formulation	Dose [mg]
			Subgroups Size		
I	Study group, F1; orally administration	30	A 6 B 6 C 6 D 6 E 6	F1	0.5
II	Study group, F2; orally administration	30		F2	1.0
III	Referential product A; orally administration	30		Reference A	1.0
IV	Referential product B; orally administration	30		Reference B	1.0
	Total:	120			

**Table 5:** Flow properties of tableting blends.

Powder Mixture	Angle of Response [°]	Hausner Ratio	Carr’s Index [%]
Physical mixture: HT, Prosolv^®^ ODT, magnesium stearate	42 (passable)	1.28 (passasble)	24.65 (passable)
Tableting mass: HT:HPMC (1:1) after granulation, Prosolv^®^ ODT, magnesium stearate	28.57 (excellent)	1.09 (excellent)	7.86 (excellent)

**Table 6:** Physicochemical characteristics of prepared ODMTs.

Parameter	Formulation	
	F1	F2
Weight [mg]	7.7 ± 0.2	7.8 ± 0.2
Thickness [mm]	2.0 ± 0.1	2.1 ± 0.1
Hardness [N]	10.5 ± 1.2	5.3 ± 0.9
Friability [%]	0.8	0.9
Drug content [mg]	0.52 ± 0.1	1.1 ± 0.1

**Table 7:** Sensory assessment of ODMTs determined using the following point scale: assessed as follows: 0—not bitter, 1—slightly bitter, 2—moderately bitter, 3—very bitter.

Formulation						
Volunteer	F5	HT 2.5 mg	F1	F2	F3	F4
1	0	3	1	2	2	2
2	0	3	2	2	3	3
3	0	3	1	2	2	3
4	0	3	0	1	2	2
5	0	3	0	1	2	2
6	0	3	1	2	2	3
7	0	3	1	2	3	2
8	0	3	0	1	2	2

**Table 8:** Bioavailability parameters of the tested formulations (F1 and F2) and reference products A and B (results shown as mean ± SD, n = 6).

Parameters	F1	F2	Ref. A	Ref. B
C_max_ [ng/mL]	50.6 ± 2.5	81 ± 2.6	85.6 ± 2.1	85.7 ± 1.6
t_max_ [h]	2	2	2	2
AUC _(0–8)_	245 ± 2	409 ± 10	412 ± 13	429 ± 7
AUC _(0→∞)_	292 ± 12	505 ± 26	470 ± 25	476 ± 18
AUC _(0–8)_/AUC _(0→∞)_	0.81 ± 0.03	0.81 ± 0.03	0.88 ± 0.03	0.9 ± 0.02
Λz	0.27 ± 0.03	0.25 ± 0.02	0.31 ± 0.03	0.36 ± 0.04
T_0.5_ [h]	2.6 ± 0.3	2.8 ± 0.3	2.2 ±. 0.3	2.0 ± 0.2
EBA_Ref.A_ [%]	59	99	-	-
EBA_Ref.B_ [%]	57	95	-	-

## Data Availability

The original contributions presented in the study are included in the article, and further inquiries can be directed to the corresponding authors.

## Supplementary Materials

The following supporting information can be downloaded at: https://www.mdpi.com/article/10.3390/pharmaceutics16081041/s1, Table S1. The dosage of HT in children, with regard to the possibility of using conventional tablets and designed ODMTs [38]. Table S2. Mean HT sample concentration (n = 6) of each sample point after oral administration of F1, F2 and reference products A and B. Table S3. HT each sample concentration of each sample point (0.5; 1; 2; 4 and 8 h) after oral administration of F1, F2 and reference products A and B.
