# Supplementary material for: Utilization of the Drug–Polymer Solid Dispersion Obtained by Ball Milling as a Taste Masking Method in the Development of Orodispersible Minitablets with Hydrocortisone in Pediatric Doses
Source: Pharmaceutics. 2024 Aug 4;16(8):1041. doi: 10.3390/pharmaceutics16081041 (PMC11359562; doi:10.3390/pharmaceutics16081041)
Supplement: Supplementary file 1 [file pharmaceutics-16-01041-s001.zip › pharmaceutics-3113155-supplementary.pdf]

Article

Supplementary Materials: Utilization of the Drug–Polymer Solid Dispersion Obtained by Ball Milling as a Taste Masking Method in the Development of Orodispersible Minitablets with Hydrocortisone in Pediatric Doses

Monika Trofimiuk, Katarzyna Olechno, Emil Trofimiuk, Anna Czajkowska-Kośnik, Patrycja Ciosek-Skibińska, Klaudia Głowacz, Joanna Lenik, Anna Basa, Halina Car and Katarzyna Winnicka

Table S1. The dosage of HT in children, with regard to the possibility of using conventional tablets and designed ODMTs [38].

**Age range**	**Single dose* [mg] (3 times per day)**	**Dosage available in conventional tablets**		The number of ODMTs given to the child (dose 0.5 mg)
	**0.13 mg/kg b.w.**			
	**0.27 mg/kg b.w.**	10 mg	20 mg	
**0- 1 month of life**	0.5-0.7	-	-	1-2
	1.0-1.5	-	-	2-3
**2-12 month of life**	0.7-1.3	-	-	1-3
	1.5-2.7	¼ tab.	-	3-5
**2-4 year**	1.7-2.3	¼ tab.	-	4-5
	3.5-4.8	½ tab.	¼ tab.	7-9
**5-7 year**	2.5-3.2	¼ tab.	¼ tab.	5-7
	5.2-6.6	½ tab.	¼ tab.	10-13

*The smallest effective single dose of HT (0.13 mg/kg of body weight and 0.27 mg/kg of body weight) administered orally were considered based on the daily dose 0.4-0.8 mg/ kg of body weight.

Table S2. Mean HT sample concentration (n=6) of each sample point after oral administration of F1, F2 and reference products A and B.

Concentration [ng/mL]	F1	F2	Ref.A	Ref.B
$C_{0.5}$	6.57 ± 0.6	11.00 ± 2.7	20.00 ± 3.0	19.87 ± 2.1
C_1	36.20 ± 1.5	63.33 ± 1.2	72.30 ± 3.4	71.54 ± 2.0
C_2	50.60 ± 2.5	81.00 ± 2.6	85.60 ± 2.2	85.70 ± 2.6
C_4	37.60 ± 1.1	62.50 ± 1.8	61.43 ± 2.0	68.25 ± 1.3
C_8	12.74 ± 1.4	23.47 ± 2.6	17.78 ± 2.6	16.73 ± 2.7

* Mean concentration of HT measured in 0.5; 1; 2; 4 and 8 h.

Table S3. HT each sample concentration of each sample point (0.5; 1; 2; 4 and 8h) after oral administration of F1, F2 and reference products A and B.

**F1**						
**Concentration [ng/mL]**	**1**	**2**	**3**	**4**	**5**	**6**
$C_{0.5}$	5.82	5.72	5.82	6.82	6.98	6.90
C_1	38.10	37.90	5.72	36.20	34.20	35.12
C_2	47.89	53.05	7.20	52.10	48.17	53.25
C_4	38.50	35.85	6.82	37.54	38.65	36.78
C_8	10.65	13.53	6.90	12.75	14.23	13.68
**F2**						
**Concentration [ng/mL]**	**1**	**2**	**3**	**4**	**5**	**6**
$C_{0.5}$	11.54	14.52	11.85	10.95	10.87	6.25
C_1	62.87	62.54	63.94	63.64	65.24	61.74
C_2	84.12	78.18	79.85	81.58	83.97	78.68
C_4	59.45	63.11	61.80	64.25	64.21	62.16
C_8	19.10	22.08	24.04	26.12	25.75	23.70
**Ref. A**						
**Concentration [ng/mL]**	**1**	**2**	**3**	**4**	**5**	**6**
$C_{0.5}$	18.85	19.41	25.58	20.07	18.15	17.25
C_1	70.56	71.84	77.74	74.85	70.24	68.54
C_2	84.98	87.88	88.23	82.79	85.27	84.46
C_4	62.45	59.15	64.64	61.52	59.75	61.08
C_8	17.87	19.65	20.51	14.23	19.34	15.09
**Ref. B**						
**Concentration [ng/mL]**	**1**	**2**	**3**	**4**	**5**	**6**
$C_{0.5}$	17.54	19.92	19.78	23.41	18.18	20.40
C_1	68.42	70.84	72.45	74.25	72.68	70.58
C_2	89.03	85.02	83.56	82.23	88.30	86.08
C_4	70.52	68.40	67.48	68.25	66,61	68.22
C_8	17.62	13.54	17.76	20.41	13,63	17.40
